# BLSTM based night-time wildfire detection from video

**DOI:** 10.1371/journal.pone.0269161

**Published:** 2022-06-03

**Authors:** Ahmet K. Agirman, Kasim Tasdemir

**Affiliations:** 1 Electrical and Computer Engineering, Abdullah Gül University, Kayseri, Turkey; 2 Computer Engineering, Abdullah Gül University, Kayseri, Turkey; Universiti Tunku Abdul Rahman, MALAYSIA

## Abstract

Distinguishing fire from non-fire objects in night videos is problematic if only spatial features are to be used. Those features are highly disrupted under low-lit environments because of several factors, such as the dynamic range limitations of the cameras. This makes the analysis of temporal behavior of night-time fire indispensable for classification. To this end, a BLSTM based night-time wildfire event detection from a video algorithm is proposed. It is shown in the experiments that the proposed algorithm attains 95.15% of accuracy when tested against a wide variety of actual recordings of night-time wildfire incidents and 23.7 ms per frame detection time. Moreover, to pave the way for more targeted solutions to this challenging problem, experiment-based thorough investigations of possible sources of incorrect predictions and discussion of the unique nature of night-time wildfire videos are presented in the paper.

## 1 Introduction

Wildfire is a significant threat worldwide and among the significant devastating natural disasters that can have immediate and long-term effects on the environment, the people, and the economy [1]. In favorable conditions, the spread speed of bushfires can attain as high as **24** km/h which makes its suppression extremely hard [2]. Therefore, one of the most crucial steps in firefighting is early detection of the fire after the ignition. As one of the most common ways of early detection, analyzing live videos for a possible wildfire helps mitigate the severity of the aftermath of forest fires, as the statistical data indicates [3, 4].

Fire videos can be categorized as daytime and night-time. Night-time wildfires have a considerable percentage among fire incidents. For instance, 20% of reported forest fires in Turkey and 48% of city fires in Istanbul occurred at night or low-light conditions from July to November 2020. Nevertheless, detection of night-time wildfires from videos has not been used effectively due to its challenges [3, 4].

There are several challenges to wildfire detection from a night-time video. Some are related to the nature of the fire, and others are more related to the camera’s limitations. Compared to its low cost, RGB cameras bring their own challenges to the task of fire detection, especially when night fires are in question.

Historically, previous studies that worked on wildfire detection with night videos employed hand-crafted features until the 2013s [5]. Since then, the paradigm has shifted from hand-crafted features to hand-crafted networks. Today, the recent approach generates features out of the networks, which has been possible by convolutional neural networks (CNN).

CNN-based methods have shown their effectiveness on object detection tasks. However, it is challenging to detect night fires from RGB cameras with well-known object detection algorithms for night-time fire detection in video. One particular reason is that it is not benefiting from the temporal relation of the frames. To alleviate this limitation, employing Recursive Neural Networks (RNNs), which can model a video as a data sequence, has been an option.

2D CNNs can be used for extracting spatial features and RNNs for extracting temporal features. Cascaded CNN+RNN structure is a well-known approach used in various fields such as video description extraction, action recognition, etc., and its effectiveness is shown in multiple studies [6]. Using both CNNs and RNNs in a pipeline has the potential to increase detection performance. This study is the first time that approach has been used against night-time wildfire detection problems to the authors’ best knowledge.

The present work proposes a two-stage approach combining spatial and temporal information of an object appearing in a night-time video. The first stage (CNN) computes spatial features, and the second stage (RNN) makes the temporal analysis depending on these features. The CNN stage employs transfer learning on a pre-trained GoogLeNet [7] to reduce the training time of the overall network. The second stage employs the bidirectional long short-term memory (BLSTM) network and is trained with feature maps obtained from the first stage for each video frame. After the network pipeline is trained, it can readily be used for detection in for example watchtowers that are equipped with CCTV cameras. The network can be deployed in two ways. First, it uses the weights determined with the initially training-test procedure and they are not updated in response to different fire or non-fire samples events. Second and the adaptive one is the pipeline continuously updates itself by simultaneous re-training iterations. By doing so, the pipeline always becomes up to date for changing environmental conditions.

As mentioned before, the night fires have a unique nature. Due to the low-lit environment and extremely bright fire objects appearing in the same scene, the physical limitations of the camera, such as the dynamic range, give a unique digital video. Investigating the typical features of night-time wildfire videos, discussing possible sources of incorrect classifications and possible solutions are essential for developing well-targeted solutions. These are also thoroughly investigated and discussed in this paper.

Therefore, the novelties and contributions of this study can be summarized as:

1. The proposed method incorporates both the spatial and temporal behavior of a night-time wildfire by using a CNN+RNN based network and detects fire at min of 23.4 ms per frame.
2. It employs BLSTM for capturing both forward and backward temporal relationships in the night-time wildfire video,
3. It uses decisions from spatial and temporal networks to employ majority voting to improve the prediction accuracy,
4. The data samples which give the most failure in night-time wildfire detection tasks are identified and carefully investigated, and the nature of night-time wildfire videos is discussed. It is revealed in CNN+BLSTM networks that a non-fire event that is seen on fire scenes has potential to suppress the fire event and revert the decision as “non-fire” instead of “fire” or vice versa.
5. A novel night-time wild, rural, suburban area fire detection dataset is proposed to push night-time video fire detection (VFD) research forward.

This paper is organized as follows: Characteristics of night-time fires and objects are described in Section 2. In Section 3, related work on night-fire detection from videos is summarized. In Section 4, the proposed method is explained. In Section 5, the experimental setup is illustrated. In Section 6, the results of the experiments are discussed, the performance of the GoogLeNet+BLSTM network is evaluated, and majority voting introduced to improve prediction performance. In Section 7, misclassifications are discussed in detail and finally in Section 8, the findings are summarized and the conclusions are drawn.

## 2 Nature of night-time fires

Night-time object detection is a challenging task compared to its daytime counterpart. It is impossible to perceive non-luminous objects with RGB cameras without a distinct light source in a dark environment. Object detection in low-light conditions is sometimes possible but challenging depending on the light source’s flux, the contrast in the scene, the objects’ morphology, reflection and distance from the camera, and the light-source type which affects contrast against background. It is difficult to distinguish objects (i.e., road signs) from the background in a low contrast image without manually enhancing it, i.e., by changing color curves as in [8].

Another challenge is the insufficiently visible texture which makes identifying objects in their surroundings difficult. When images are blurry or in an indistinguishable texture due to the camera, heavy smoke, or fog in the environment, then CNN filters will generate similar feature maps. For example, an image of a fire object around a house with a significantly reduced texture and an image on a vehicle headlight in a heavy smokey environment will have significantly reduced textures as in [9]. As a result, it will mislead the network to an incorrect prediction.

Other than the high International Organization for Standardization (ISO, sensitivity of a camera to light)’s contribution, the dark images naturally contain a considerable amount of noise as a result of diffused smoke in the air as in [10]. This noise also brings a challenge to the training process.

Night-time images contain minimal color information compared to daytime images. This makes them very close to binary images; thus, color and texture analysis becomes harder [11]. Smoke detection is possible with daytime videos. However, due to the lack of rich color information, this is impossible primarily for night-time videos. Cameras have a limited dynamic range. When the parameters such as exposure and ISO are set for the foreground object region, the remaining part of the scene becomes near black.

Moreover, the light sources visible in a frame introduce further challenges due to the camera’s dynamic range shift. Those effects are explained in the experimental results section.

In some cases, the scene includes only bright or dark regions making the video akin to a binary video. Binary images give limited information about an object, including its shape and position in the frame. It lacks color and texture information which are central in object detection tasks. For example, a lantern or a freshly ignited flame might look indistinguishable in night images.

Nonetheless, a binary video offers descriptive clues about the investigated object, such as its motion behavior throughout the video. In night-fire videos, the flame has a distinct motion behavior such as flickering, shooting high into the air, dying down or flaring up, and temporal disappearance due to smoke occlusion. Therefore, this study aims to benefit from these temporal behavioral characteristics of a fire object.

## 3 Related work

Fire detection from videos has been studied by many research groups [5, 12–15]. Recent work [16] gives a comprehensive review of flame and smoke detection from videos. However, the majority of the related studies target daytime images and videos. There are also a limited number of works targeting the night-fire detection problem [17–22]. The most relevant studies are briefly explained below.

Gunay et al. proposed a set of hand-crafted features for night-fire detection [17]. They developed a decision-making system that fuses decisions of sub-algorithms. These sub-algorithms make decisions based on detecting slow-moving objects, bright regions, periodic regions, and moving region interpretation. Ho and Chen used a CCD camera and a laser light to detect smoke at night [18]. They analyzed spectral, diffusing, and scattering characteristics of the trajectory of the laser beam with a fuzzy reasoning system to detect fire smokes. Gomes et al. proposed a rule-based fire detection system tested on night-time fires besides indoors, rural, and urban fires [19]. They used two parallel working pipelines, one for fire detection and the other for fire confirmation, to make the final fire decision. Agirman and Tasdemir proposed an SVM-based method for short to mid-range night-fire detection. Their method first extracts spatio-temporal features of several consecutive frames and classifies the temporal behaviors of the objects with SVM [20].

Park and Ko proposed a multi-staged night-time fire classification method using a modified YOLOv3 architecture and Random Forests (RF) [21]. They first analyzed the videos with ELASTIC-YOLOv3 to detect candidate fire regions per frame. Then, they generated fire tubes from fire frames based on a rule that joins fire object candidates in successive frames. They generated a histogram of oriented features (HoF) from the fire tubes, then transferred them to a bag of features (BoF) with a code-book mapping. Finally, they used a bag of feature histograms as features to train an RF classifier. In the process of fire tube generation, a threshold that allows adding a frame to the fire tube needs to be manually set. This threshold should be adjusted according to the distance between the fire object and the camera. A dataset containing a diverse set of real-world examples is not practical to decide on a global threshold covering all samples. In addition, they extract a histogram of features from each frame of an object tube. The motion behavior of the flame, such as flickering, cannot be captured because the indexes of frames are lost when the features are put into a bag of features set. The limited generalization capacity of the method makes it suitable only for scenarios where the dataset distribution is not diverse.

Pan et al. developed a pruned CNN via Fourier analysis to detect wildfire and tested its performance on a limited number of fire videos besides daytime videos [22]. They used MobileNetv2 and pruned redundant low-energy kernels and similar kernel pairs by calculating their DFTs, thus letting them save approximately 7% time and 22% storage.

In its problem nature, fire detection is a subdomain of object detection. Evaluating performance of a network pipeline in object detection, a well-defined set of metrics based on a ground truth method should be employed. Two domains of ground-truths can be defined for fire datasets. Spatial ground-truths are generated at pixel-, region-, or frame-level. A pixel-level ground-truth identifies the label of each pixel in a frame. Therefore, it gives the densest ground-truth information about a frame. However, it doesn’t give any neighboring information between pixels. A region-level ground truth refers to a region of interest where a certain area of the frame is labeled as positive or negative. It can still divide the entire frame pixels as positive or negative and can give neighboring information between cells. A frame-level ground-truth implies that the target object is contained in all pixels of the frame. Temporal ground-truths, on the other hand, are generated at frame-, interval-, or video-level. A frame-level temporal ground-truth implies which frame at what time instance contains the target object, and an interval-level ground-truth implies the target object is contained at all frames in a certain interval of the video. It is noted that none of the temporal ground truths can give spatial information about the labeling. For example, let a 10-seconds video contains fire objects at only 2nd to 4th seconds, then only this interval is labeled as fire. Finally, a video-level temporal ground truth implies that each frame of the video contains the target object, then the video is labeled as fire or none of them contains the target object, then the video is labeled as none fire.

Ground-truth depth and domain is important for the method used in fire detection. For example, consider that a video-level temporal ground-truth of a video is fire; however, in the same video, some of the frames do not contain a fire object. Also consider that a temporal deep learning method will be used for the analysis. Then it should be taken into account that the algorithm will also learn from the frames without fire as if they are fire and this will affect the training process.

Depending on ground-truth type employed for the data, a performance metric should be selected. In [23], authors give a comprehensive review of spatial ground-truth performance metrics based on intersection over union (IOU) for both images and videos. With IOU, a bounding box or a closed boundary line around a target object is required to calculate intersection and union of actual bounding box (or blob generated by the closed boundary line) and predicted bounding box (or blob). In the case of temporal ground-truthed data, there are no bounding boxes or boundary lines. In that case, IOU is not suitable, and the whole image or video is considered to belong to a class. Accuracy and F1 scores are two useful performance metrics chosen for our experiments as also by recent VFD studies including [19, 21, 22].

## 4 The proposed method

Distinguishing fire from non-fire objects in night videos is problematic if only spatial features are to be used. Those features are highly disrupted under low-lit environments because of the physical limitations of the camera and other reasons, as discussed in Section 2. This makes the temporal behavior of the bright object indispensable for classification.

To capture the temporal behavior of a fire object along with its spatial features, a coupled spatio-temporal behavior analysis is crucial. To this end, a spatio-temporal network structure consisting of CNN and RNN is proposed. The proposed network first extracts the spatial features of fire candidate videos of various lengths with the help of a pre-trained GoogLeNet CNN network, as explained in Section 4.1. Second, temporal learning is performed using a BLSTM RNN network, as explained in Section 4.2. In Section 4.3, the cascaded CNN+RNN model is demonstrated.

### 4.1 The first stage: Spatial feature extraction

The first stage of the proposed network is spatial feature extraction. Since, the detection of fire will be conducted on sequences of images; the model should be able to process image data and obtain spatial characteristics that will be essential in understanding objects in a scene.

CNNs are the neural network models that can work on images, learn from them, and execute desired deep learning tasks, i.e., object detection, image segmentation, image classification, etc. In general terms, a CNN consists of an input layer, a number of hidden layers, and classification layers. In the input layer, an (*H* × *W* × 3) RGB image is given as an input, where *H*, *W*, and 3 is the height, the width, and the number of channels of the image, while preserving its spatial grid-like structure.

In the hidden layers, one important layer type is the convolutional layer. This layer receives an input image and scans a (*k* × *k* × 3) filter over it which is termed as convolution process. The filter and its projection onto the image matrix are element-wise multiplied then summed to get a weighted sum. Values of elements of the filter are termed as weights which should be optimized during training. Convolution process generates an (*M* × *N*) convoluted matrix which is input to an activation layer. In the activation layer, an activation function is applied element-wise to the convoluted matrix to determine which cells to fire. A common activation function is RELU among many others and is required to make negative elements of convoluted matrix zero and add non-linearity to the network. The activation process generates an (*M* × *N*) activation or feature map which is then input to a pooling layer. A pooling layer summarizes the most important information in a feature map. A common pooling method is max pooling which gets the max value of a projection sub-matrix onto the feature map and finally generates an (*m* × *n*) matrix. This convolution, activation, pooling sequence can take place numerous times depending on the desired architecture which, in general, constitutes depth of the hidden layers.

Assuming the final output of the hidden layers is an (*m* × *n*) matrix, the first layer of classification layer, i.e., flatten layer, converts it to an (1 × *mn*) vector to make it a useful input to a conventional multi-layer perceptron (MLP). Final layer values of MLP are passed to a softmax layer which is required to compute probabilities of each label that the input image belongs to. An argmax function finally picks the max probability class among others and delivers a prediction for the class of the image with the corresponding probability.

A pre-trained GoogLeNet architecture is picked for spatial feature extraction. The GoogLeNet architecture set a new state of the art for object detection in the ImageNet Large-Scale Visual Recognition Challenge 2014 (ILSVRC 2014). In this work, the network was pre-trained on the ImageNet [24] and is available from MATLAB^®^.

GoogLeNet has been used in previous studies [25]. Their results show that it has a high detection accuracy of 96.7% on a subset of ImageNet with flame, smoke and other flame-like labels. In another study, GoogLeNet’s performance is tested and compared with other well-known models [26]. It reports that GoogLeNet attains 99% accuracy, which is the highest among AlexNet, VGG13, and modifications of them when tested on wildfire videos taken from UAVs. Researchers designed their own models inspired by GoogLeNet because it has higher accuracy than models like AlexNet and is easily adaptable to field-programmable gate array (FPGA) platforms [27, 28]. They received a 94.43% on BowFire and MIVIA Fire Detection datasets and 93% accuracy on Furg Fire Dataset, respectively, with their modified GoogLeNet network on respective fire detection video datasets. Finally, it was reported that Inception-v3 leads to 2.5% more computational cost than GoogLeNet (Inception-v1) [29]. With the findings on day-time fire datasets mentioned above in the literature and extensive performance comparisons [30], it is conjectured that GoogLeNet is a reasonable choice for less computational complexity, less model complexity, and relatively high accuracy.

GoogLeNet approximates dense layers by employing local sparse units. These units (Inception modules) can be repeated spatially in the architecture. An Inception module receives input from a previous layer, processes convolutional layers with different kernels in a parallel fashion, and concatenates all parallel outputs depth-wise into one tensor. To reduce network resolution, max-pooling layers are used. The Average-pooling layer is used instead of an extra fully connected layer, leading to additional over-fitting [31].

**Fig 1:**
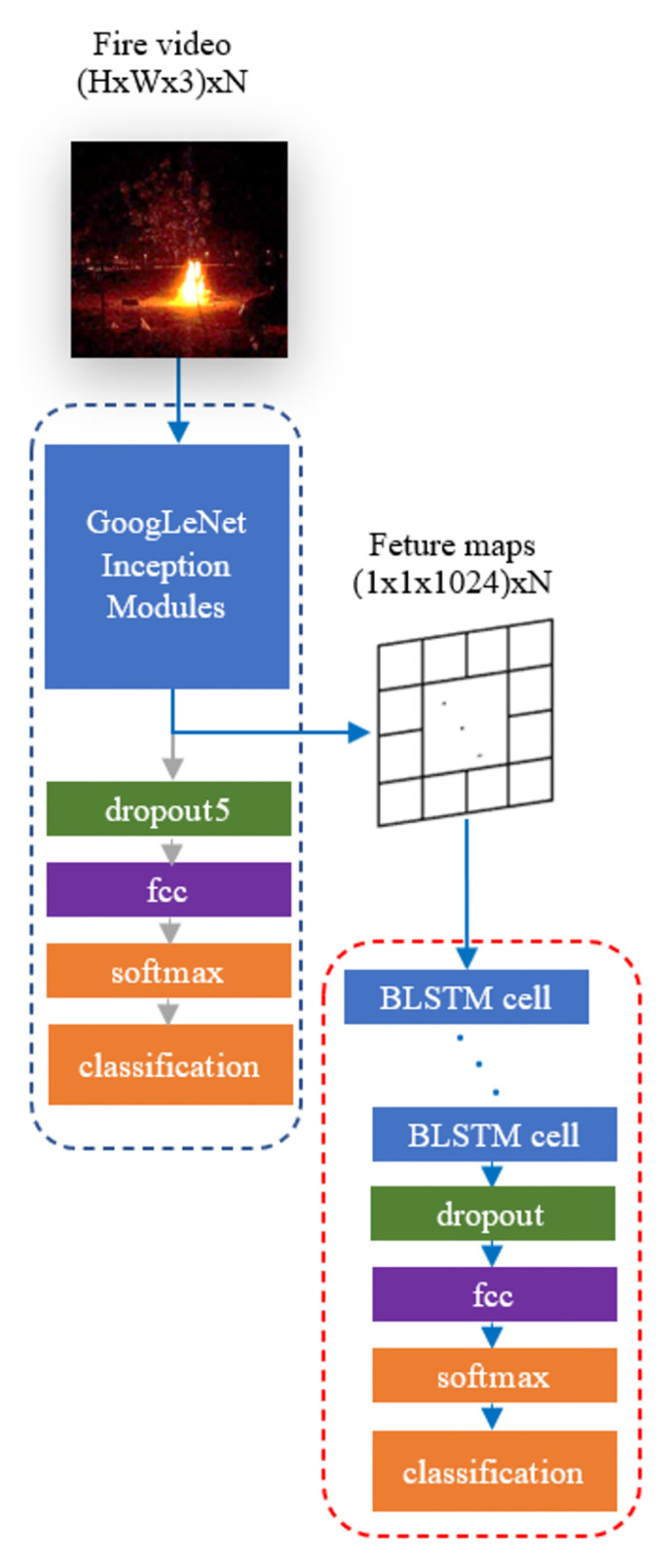
GoogLeNet+BLSTM architecture. In the dashed blue box, a standard GoogLeNet network structure is shown. GoogLeNet receives images or sequences of images in 224 × 224 size. In a dashed red box, a standard stacked BLSTM network structure is shown in rolled form. A BLSTM network receives a series of data in 1024 × 1 size. If *N* number of such vectors is the case, then input is in 1024 × *N* size. Blue arrows starting from the input video and ending at the classification box show the flow diagram of the proposed method.

We implement transfer learning with the GoogLeNet network that is pre-trained on ImageNet. Each frame is fed to the pre-trained GoogLeNet CNN network, and a corresponding feature map is extracted from the final average pooling layer (Fig 1). Thus, a video, as an image sequence of size *H* × *W* × 3 × *N*, is converted to a tensor of size (1 × 1 × 1024) × *N* where *H*, *W*, and *N* is the height, the width, and the number of frames of the video, respectively. The sequence of these vectors is used for further temporal analysis, using the BLSTM network [32], as explained in the following section.

### 4.2 Temporal analysis

Long short-term memory (LSTM) is a special kind of recurrent neural network that can learn from sequentially related data without losing essential features throughout time [33]. In other words, LSTM networks can learn from past events and use this knowledge to classify present events. In order to keep track of the past, it requires a useful summary of the past carried to the present. This is accomplished by an updating cell state also termed as long-term memory. The long term memory is updated by dropping insignificant information and keeping the significant one by distinct internal neural networks. There is another state known as hidden state and is required to update short-term memory and generate an output prediction. Short-term memory is also obtained by another internal neural network. In the end, the LSTM network makes predictions for a given input by keeping track of long-term and short-term ‘past experience’.

This property makes them a prominent candidate for video captioning [34]. The building block of an LSTM network is a cell engine that receives input of the current time step along with the cell state and output of the previous time step (hidden state) to generate the current cell state and output. Then these are fed to the next cell iteration.

LSTM is also applied to daytime fire detection problems in [35, 36]. The obtained accuracies are 97.92% and 93.3%, respectively.

However, LSTM models require a longer training time than CNN models since they cannot be run in parallel. On the other hand, LSTM architecture can infer results only in a feed-forward direction, i.e., from past to present. It cannot generalize predictions with the valuable knowledge from the ‘future.’ For example, when an LSTM network is to predict the next word of the statement “I like to make …”, there are numerous options to choose from. However, if the network were to have a subsequent statement of “I believe melodies heal people” then the prediction would likely be “music” rather than i.e., “cake”. In the similar way, predicting the fire events in a video not only via the past experience but also with respect to knowledge of the “future” can be accomplished by a bidirectional LSTM (BLSTM) [37].

Considering that the night-time fire video scenes are inadequate in terms of spatial features compared to daytime videos, the contribution of temporal features to the decision process becomes increasingly important. To capture the temporal behavior of the candidate object and its advantages over LSTM, the BLSTM network is used as the second stage of the proposed method.

#### 4.2.1 Second stage: BLSTM

**Fig 2:**
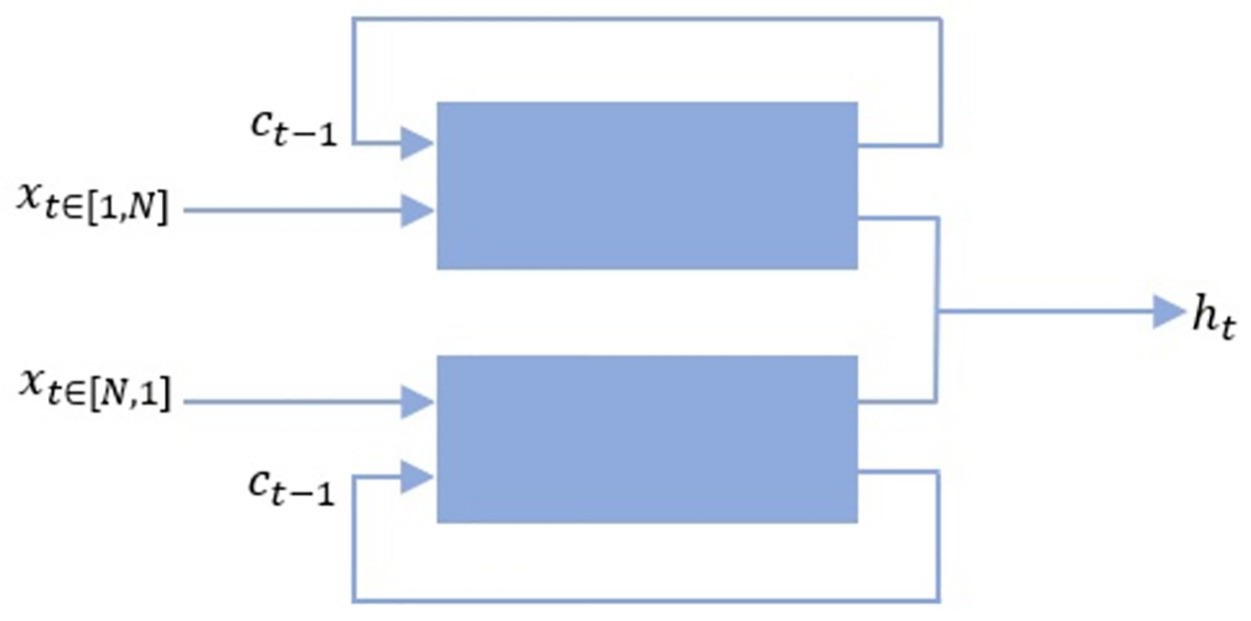
BLSTM cell engine.

A BLSTM cell uses two coupled LSTM cell engines, as summarized in Fig 2. The coupled engine receives the first elements of a sequence and time-reversed version of it, and then produces an output. This procedure continues until all elements of (sequence, reversed version) pairs are processed by the engine. This allows the network to learn both from past and ‘future’ simultaneously and gives more accurate results in classifying a scene.

CNN features extracted from the first stage are fed to a BLSTM network stack for training. The rolled network structure is given in Fig 1. The BLSTM stack receives each sequence with the size 1024 × *N* where *N* is the number of frames per sample video.

Stacked BLSTM performs better than single-cell counterparts in accuracy, ability to learn at different time scales, and ability to manipulate parameters with increased non-linear operations [38]. A dropout layer is required to avoid overfitting for long stacks. Final conventional layers are a fully connected layer output of two for (fire, non-fire) classes, a softmax layer for probability computations, and a classification layer for cross-entropy loss computations.

### 4.3 Model architecture and testing

Training the overall network normally includes training of two sub-networks: CNN and BLSTM. However, when it is available, adopting a pre-trained network is useful in order to reduce overall network training time and obtain a working final classifier as soon as possible. In Section 4.1, it is mentioned that a pre-trained model is adopted only for the CNN model, which is also termed as transfer learning. Therefore, spatial feature extraction is obtained by using a pre-trained CNN network given in Fig 1. In the figure, the full-stack trained CNN network receives never-seen images and generates a prediction in the end. In this work, the full stack trained CNN is not used as is, in fact is only used to extract features of the video frames from the final pooling layer’s output of the GoogLeNet network. This implies that final dropout5, FCC, and softmax layers are excluded from the overall classifier. In Section 4.2, these features are used to train a BLSTM network given in Fig 1.

Finally, these two stages are connected to each other, as shown in Fig 1. Connecting the two stages requires two trained models adapted to a pipeline model with the following steps. First, since the pipeline will receive videos, the image input layer of CNN is replaced by a sequence input layer and input videos are converted from sequences of frames to a tensor of images to let the CNN convolutional layers receive video data image by image. Second, CNN is not expected to output predictions but only generate feature maps; as a result, dropout5, fully connected, softmax, and classification layers are unnecessary and truncated from the CNN structure letting the last CNN layer be pool5 which is the final global average pooling layer of GoogLeNet. Third, the pool5 layer will be the input layer of the BLSTM architecture, so the input layer of the BLSTM layer is dropped and the remaining structure is kept as it is. Finally, the adjusted and truncated CNN is connected to truncated BLSTM to obtain the end-to-end classifier pipeline.

In summary, an *N* frame-long video of size *H* × *W* × 3 is given as an input to the first layer in the figure. The data is processed through the CNN layers until the average pooling layer. Here, the final feature maps are generated as a 1024 × *N* tensor, and it is fed as input to the first cell of the stacked BLSTM layers. The fully connected final layer outputs the probabilities of the two classes. The class with the highest probability in the softmax layer is finally labeled to be fire or non-fire.

### 4.4 Dataset

Well designed datasets are a backbone stage of developing automatic fire detection systems based on computer vision and machine learning. To the best knowledge of the authors, Neal et al. published the first work on image based fire detection with neural networks in 1991 [39]. Since then, there have been over 3 hundred research works on the problem of VFD. These publications used image or video data to evaluate performance of their models. The data of the majority of these works are not accessible due to lack of access links, broken links, inaccessible links from other countries, etc. Even though some researchers created their own dataset by combining data from other accessible known datasets, access links to the final dataset are mostly not given in those works. Therefore, it is not possible to replicate these methods with their original data. Papers with open access data either include direct working links or controlled access by registering to the database or signing a license agreement of the providing institution. Since the data is available, it is possible to replicate the original work with the corresponding data.

The most used dataset for model development and comparison in the literature is VisiFire dataset [40] from Bilkent University, Turkey. It includes 14 positive fire videos, 23 positive smoke videos and 2 negative smoke videos. Only 2 of these videos are negative night videos. The MIVIA Fire Detection Dataset from University of Salerno, Italy [41] includes 14 positive & 17 negative fire videos and 149 positive smoke videos including no night-time videos. Video smoke detection (VSD) dataset from University of Science and Technology of China includes 3 positive smoke videos, 3 negative smoke videos with no night-time video. The ViSOR dataset from University of Modena and Reggio Emilia, Italy [42] includes 14 positive smoke videos among other provided sets. FIRESENSE dataset [43] includes 11 positive fire videos, as well as 16 negative fire videos two of which are night-time videos, 13 positive smoke videos, and 9 negative smoke videos. FiSmo-FireVid dataset from University of Sao Paulo, Brazil [44] and the RESCUER project [45] includes 27 positive fire videos only one being night-time fire video. Furg Fire dataset from Federal University of Rio Grande, Brazil [46] contains 17 positive fire videos and 6 negative fire videos without night-time data.

Park et al. developed a new night-fire dataset which contains 10 positive and 10 negative fire videos collected from both KMU dataset and from YouTube. There is no access link to this dataset as for now to give detailed information about nature of the videos, however from their work at [21] we deduce that the positive and negative video events belong to urban areas.

In this work, a novel video dataset is created in response to scarcity of data sets particularly prepared for night-time fire events occurring at wild, rural, or suburban areas. The dataset contains night-fire videos collected from a number of public online video services for VFD research purposes.

Popular fire data collection sources are public video sharing services like Google, YouTube, Vimeo, or data repositories that are specifically constructed to hold desired fire data. Such specific fire data stores have already been mentioned in this section.

The initial step of preparing a novel dataset starts with data retrieval. A combination of search terms like fire, flame, night, wildfire, forest fire, disaster, night drive, lightning, firework, headlight, animation, flicker, etc., was used in search engines and video services to list candidate data. After beginning the search, automatic suggestions have been very helpful in accessing more useful and diverse data. It is important to note that searching for not only fire videos but also fire-like videos is crucial in creating a challenging dataset that can represent real life situations. Furthermore, videos of hand-made fires were discarded to let the algorithms trained on complex fire scenarios rather than comparatively simple experimental environments.

Each candidate video was evaluated in terms of validity, quality, and variety to determine its potential contribution to the VFD research. Data validity includes checking if the candidate data is a video (i.e., not a GIF file) with any video format, has at least 20 frames, recording time is at night or almost at night with heavy smoke for fire videos and any time for deceptive non-fire videos, includes videos from natural fire events for fire class and includes videos strongly from fire events and fire-like deceptive events for non-fire class, and is not duplicate. Data quality includes checking if the candidate data preferably has a higher resolution with a corresponding image quality. For example, old recorder videos may have higher resolutions but bad image quality, i.e., insufficient details of colors and texture, or pixelated video, etc. Choosing RGB videos contrary to black and white or grayscale videos is also considered under video quality check. Data variety aims selecting various types of fire scenarios and scenes while accepting a video candidate. High variety in terms of fire scenarios and scenes will contribute to developing more robust fire detecting pipelines. Table 1 gives the checklist for the data variety.

**Table 1:** Video tags used for the dataset.

Caption Group	Captions
Object in fire	tree fire, brush fire, forest fire, vehicle fire, exterior building fire, interior building fire, structure fire
Other light sources	head light, city light, road light, hand-held light, moon light, lightning
Objects in scene	fire truck, other vehicle, fire fighter, reporter, other people, pole
Events in scene	structure collapse, tree collapse, vehicle pass, human movement
Fire contour	Ground view: V shape, Λshape, / shape, \shape; Aerial view: S shape, C shape, water drop, free line
View of fire	aerial view, ground view, direct view, vehicle-drive view, indirect view (through car/building windows)
Camera motion	stable, include waggling, include tilts, include displacements, include zooms
Record time	day, night, semi (heavy smoke like night)
Distance	macro, short-range, mid-range, far-range

The publicly available videos are mostly made available by news channels on their accounts at YouTube; therefore they include many unwanted video fragments which should be handled at preprocessing step. All fire scenes were chosen from real-life fire incidents from 2013 to 2019. During data collection, videos were organized in terms of the incident country, location in the country, fire name, and incident time as much as possible to keep track of incident origins and prevent duplicate samples.

Creating the proposed dataset required several data preprocessing steps. The raw data collected from open sources in. mp4 format was generally not suitable to be readily used in model development. Thus, the data was cleaned from unwanted data fragments by cropping, trimming, cutting, resizing, removing duplicates, etc. The software tool used for this purpose was Adobe Premiere Pro^®^. The accepted data is then stored in. mp4 format with H.264 compression codec. Furthermore, the video data was organized for each video as either containing fire in all of the frames or none of the frames. This lets all frames of a video be labeled as either fire or non-fire and prevent the network from getting affected from counter labels during learning about a certain label. For example, a fire labeled video cannot contribute to a non-fire training process since the fire video contains the fire event in all frames. After completing these steps, all videos were added to the dataset in common video formats. In order to reduce computer work and accelerate analysis, a mat file version of the video files were created.

The dataset includes various human-interpreted captions. For instance, videos are captioned in terms of objects of interest that are being burned, such as tree fire, brush fire, forest fire, vehicle fire, exterior building fire, interior building fire, and structure fire. Another example includes captions in camera movement, such as stable, including waggling, tilts, displacements, or zooms. These captions are summarized in Table 1.

**Fig 3:**
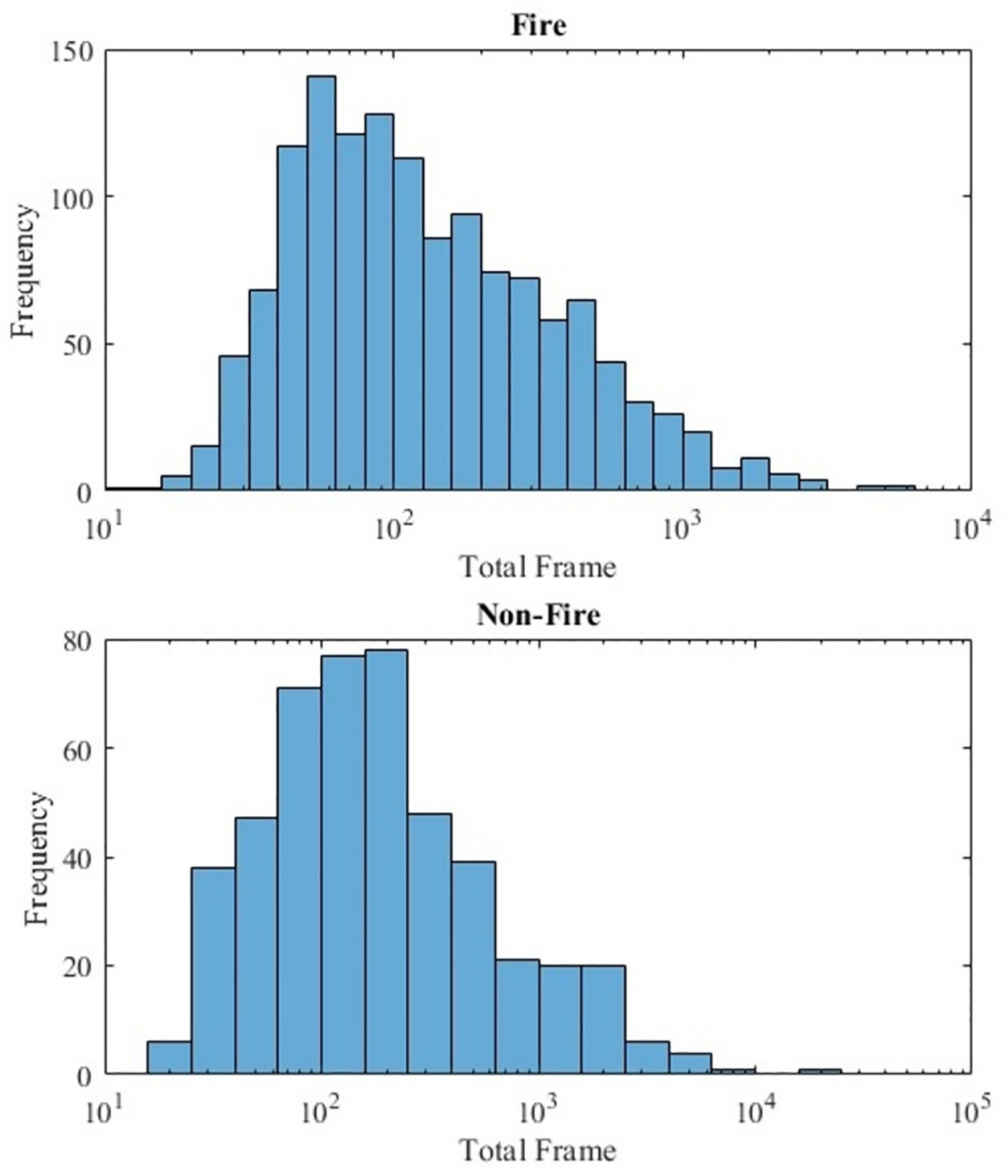
A log-scale distribution of the number of frames in both fire and non-fire videos.

Furthermore, the videos are classified in terms of difficulty (difficult, not-difficult), recording angle (same scene, same angle, same scene, different angle) to improve dataset selection. When a video can be classified as a fire event at first glance by a data operator, it is considered as not-difficult. However, when the data operator is not sure about the event at the first look and has to further evaluate it, then it is considered as difficult. A couple of daytime videos exist only for non-fire videos where tanker aircraft deploy fire extinguisher materials to the land area. They are added to challenge the network with fire-like objects. The distances are considered as follows: If a person can reach the fire area with a couple of steps, it is labeled a short-range fire. If the fire is very far away, such as a video recorded from a lookout tower, it is considered far-range. If the fire is recorded with a macro zooming and only flame structure is clearly visible, it is considered macro. Other than these, fires are considered mid-range. In total, 1835 videos comprise 1358 night-fire and 477 non-fire videos in the base dataset. Log-scale histogram charts that show frame number frequency of the videos are given in Fig 3. 90% of the videos are in 720x1080 resolution and minimum resolution (240x432) videos are only 2.2% of the dataset.

## 5 Experimental setup

### 5.1 Preprocessing

**Fig 4:**
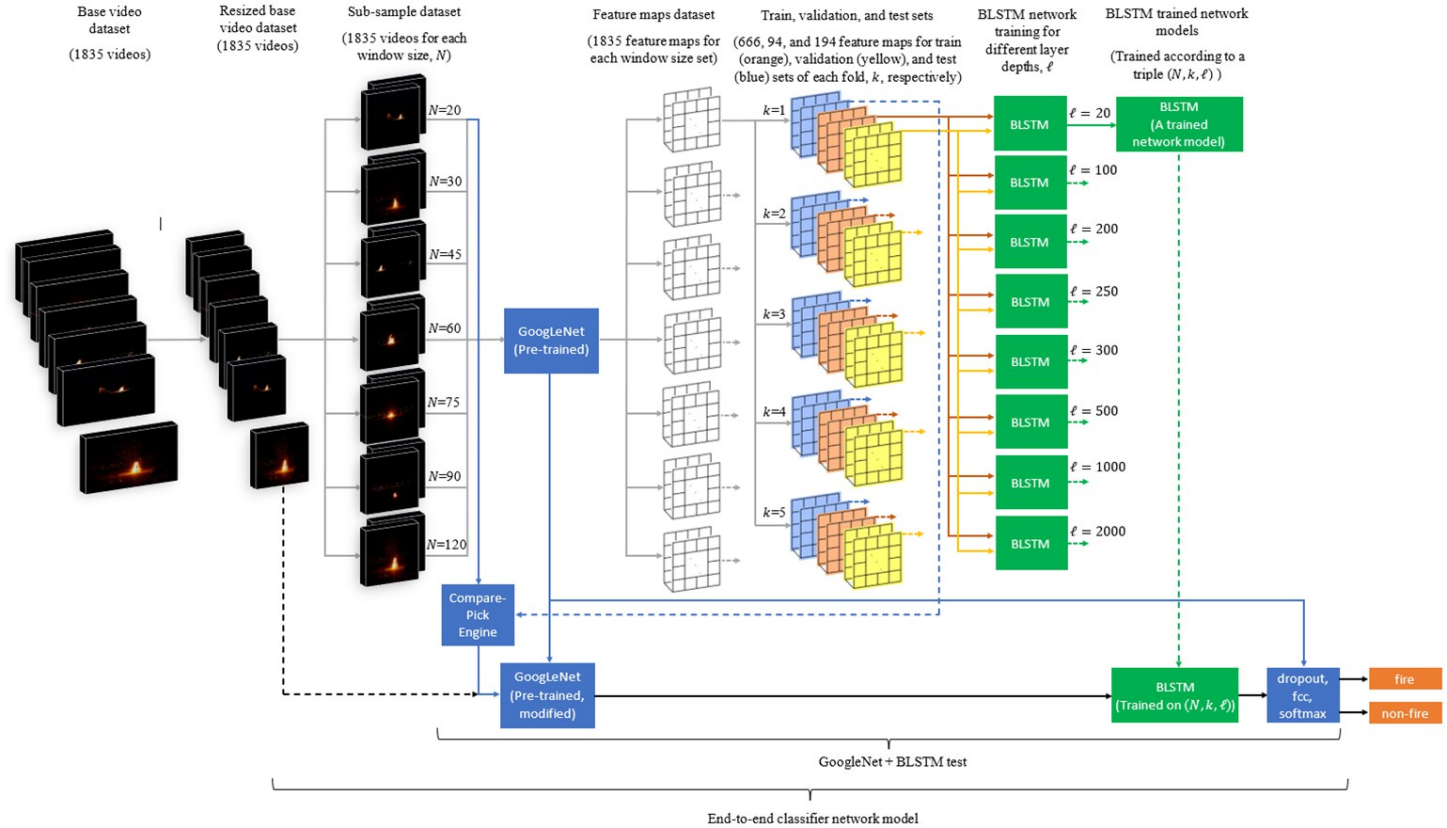
Stages of the proposed method.

In the preprocessing step, the data is organized for network training and test. Steps performed in the preprocessing step are shown with gray arrows in Fig 4.

Since base videos are in various sizes and a CNN network accepts the input in only a specific size, all fire and non-fire RGB video frames are resized to 224 × 224 × 3. This yields the resized base dataset with 1835 videos, each of which is the size of 224 × 224 × 3 × *N*.

We repeated the experiments for various video lengths to investigate the proposed method’s fire detection speed and accuracy. We name the video lengths as window size, *N*, referring to the number of frames in the temporal window. Since the detection speed from 2/3 to 2 seconds suffices for near real-time detection, smaller temporal window sizes at around 60 frames are preferable in a practical sense.

The base videos are sub-sampled with various time windows, *N*. Assuming that the videos are in 30 fps, the video length, *N* ∈ ***N***, ***N*** = {20, 30, 45, 60, 75, 90, 120}, would give 2/3 to 4 seconds detection latency. We picked max *N* sequential frames for a window size, *N*, starting from a randomly determined time position in each base video. In this way, we construct a new intermediate dataset with 1835 shorter videos corresponding to the window size, *N*. From each base video, only one sub-sample is extracted to ensure that BLSTM blocks do not memorize the similar scenes that belong to the same base video. This random subsample dataset generation step is repeated for each fold in the experiments. That is, for each fold, a new intermediate sub-sample dataset is generated randomly.

As mentioned in Section 4, A CNN network is not trained from scratch, and a pre-trained GoogLeNet architecture is used. The pre-trained GoogLeNet network model was used on the sub-sample dataset for spatial feature extraction. Thus, each video yielded a feature map of size 1024 × *N*. This map was used in the following step, the BLSTM network.

We trained a BLSTM network with a feature map set constructed for each given *N* window size. We repeated the same training process for *k* = (1, 2, …, 5) fold for each *N* and network settings. Given a window size *N*, train, validation, and test sets are generated randomly from the corresponding feature maps for each fold *k*.

There are 477 non-fire negative videos in the base video set. To have balanced positive and negative samples in both train and test sets, we picked 477 feature maps randomly out of 1358 positive samples. This constituted randomly picked 477 fire and 477 non-fire intermediate feature maps set for each (*k*, *N*) pair. The intermediate feature maps set was randomly split into three disjoint sets: 70% for training, 10% for validation during training, and 20% for testing. It should be noted that test, train, and validation sets are taken from completely different scenes. If a sample video taken from a base video is in the train set, another sample video taken from the same base video at different intervals cannot be in any of the train, the validation, or the test sets. In this way, we have a more reliable testing scenario because the train and test sets have entirely different scenes. To this end, for each (*k*, *N*) pair, 35 = |***N***| × **max**(*k*) intermediate datasets each of which contains its own training, validation, and test sets are constructed (See Fig 4).

### 5.2 Model construction and experiments

This method requires CNN and BLSTM parts trained separately, and then the pre-trained networks are concatenated to construct an end-to-end classifier network. Since a pre-trained GoogLeNet network is used instead of training the CNN from scratch, the only part left to be trained is the BLSTM network based on the extracted features from the pre-trained CNN (Fig 4). These feature sets taken from the CNN are given as input to the BLSTM block for temporal behavior analysis.

The experiments are conducted in the MATLAB^®^ environment on the Intel^®^ Xeon^®^ CPU E5-2620v2 2x2.1GHz 96GB memory hardware set.

The experiments are performed for various BLSTM network depths, *ℓ* ∈ ***L***, ***L*** = {20, 100, 200, 250, 300, 500, 1000, 2000}, on train, validation, and test sets. Moreover, each of these experiments repeated for various window sizes *N* for *k* fold. During the training, the batch size is set to be 16, and the dropout rate is 50% to prevent overfitting. The training initially continued for 30 epochs at the first three folds. In these experiments, it is observed that no improvement occurred in validation accuracy and loss after 12 epochs, i.e., the accuracy and loss graphs stalled. Therefore, to save training time, we decided that the initial setup of 30 epoch is not a good fit. and 12 epochs would suffice for the remaining experiments. Additionally, after experimenting with greater learning rate values, 0.0005 and 0.001, the learning rate was finally set to 0.0001. Since our dataset has large and many data samples, it was important to conduct experiments in viable time and computing power without conceding accuracy; therefore, Adam optimizer was chosen rather than SGDM with recommended parameters in [47]. In RNN network training, gradients can easily explode to unstable values which require limiting gradients not exceeding a threshold. The threshold value for gradients is set to be 2. In the experiments, there were 666 training samples per a training session with a batch size of 16. This makes 10 samples out of 41 batches if batches would not be shuffled after each epoch. In order to make the network see all training samples and prevent the network stuck in a local minimum, batches were shuffled at every epoch. After a training session is completed for an epoch, a validation was performed on the validation set.

In short, we investigated the effect of window size and network depth on various performance scores amounting to 56 = |***L***| × |***N***| test scenarios, *s*_*i*_ as given in Eq 1, by selecting sub-sample video lengths from 20 to 120 frames and network depth from 20 to 2000 stacked cells. Each scenario was tested with the same hyper-parameters, optimized with a smaller video set in the previous steps.

$$\begin{array}{c} s_{i}\in S,S={\left\{\left(\ell_{i},N_{i}\right)\right\}}_{i=1}^{\left|L|\times |N\right|} \end{array}$$ (1)

**Fig 5:**
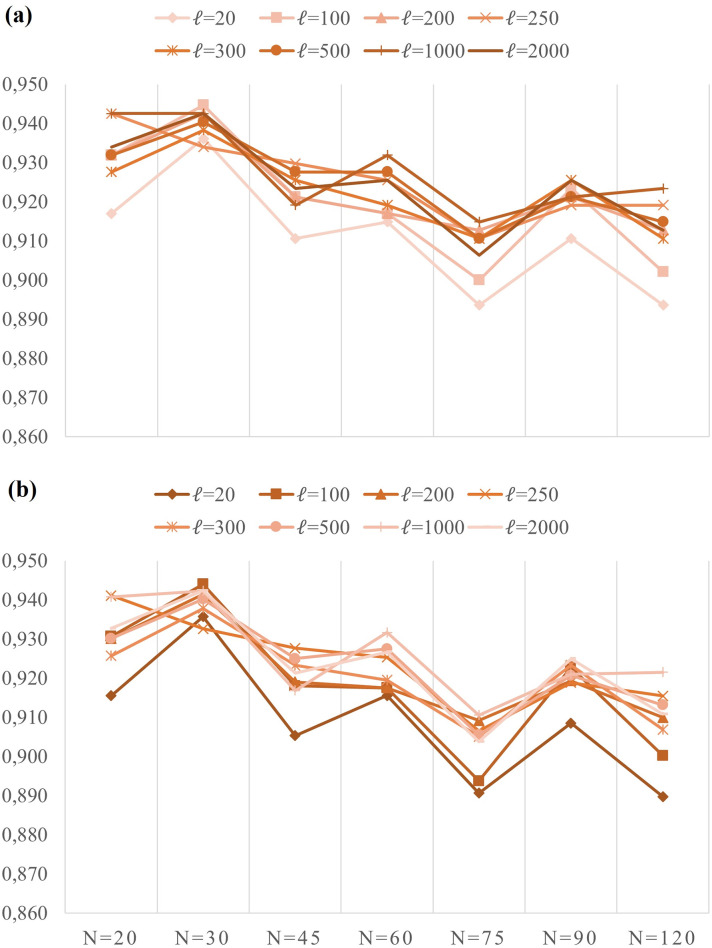
5-fold average results of validation sets (a) Accuracy (b) F1 score.

Then the scores for the experimented scenario, *s*_*i*_, are obtained by averaging the five-fold validation results. The plots of 5-fold average accuracy values and F1 scores for all scenarios are given in Fig 5. The average accuracy and F1 score of all scenarios are 92.2% and 92.1%, respectively. The maximum values of 5-fold average validation accuracy and F1 scores have been 94.5% and 94.4%, respectively, observed at *N*=30 and *ℓ*=100. The test results in Fig 5 shows that deep layers and long video samples do not contribute to training more than shallow layers and short videos.

## 6 Test results

To show the proposed network’s performance, trained models should be tested on never-seen videos in an end-to-end fashion.

These trained classifier models are generated for 7 = |***N***| different window sizes, 8 = |***L***| different layer depths, and for each (*N*, *ℓ*) pair, a 5-fold training is performed. That gives 7 × 8 × 5 = 280 models to be trained and tested. After generating and training 280 classifier models for each (*N*, *ℓ*, *k*) triple, the models are tested against a never-seen night-time fire video data set. A selection of videos for early detection results on the test set can be watched on [48]. At the 6th to 12th seconds in [48], reflection of light from an object, diffused street and headlights, and firefighters with their spatial and temporal behavior were influential in mispredictions.

The test dataset used for each model is different from that of the training and validation sets because of the random selection, as illustrated in Fig 4. The randomly selected test set used in each fold contains 194 video clips. Each 280 trained classifier model is tested with 194 respective videos, which makes 280 × 194 = 54320 predictions.

As mentioned in Section 3, we employed accuracy and F1 measure as the performance metrics. In the case of unbalanced data, i.e., the number of positive and negative samples are not equal, F1 score is a good indicator for network performance. Given that true positive (TP) is “predicted positive is also actual positive”, true negative (TN) is “predicted negative is also actual negative”, false positive (FP) is “predicted positive is in fact actual negative”, and false negative (FN) is “predicted negative is in fact actual positive” [49]; accuracy and F1 score are defined as

$$\begin{array}{c} Accuracy=\frac{TP+TN}{TP+TN+FP+FN} \end{array}$$ (2)

$$\begin{array}{c} F1=\frac{2*Precision*Recall}{Precision+Recall} \end{array}$$ (3)

where precision and recall [50] are defined as

$$\begin{array}{c} Precision=\frac{TP}{TP+FP} \end{array}$$ (4)

$$\begin{array}{c} Recall=\frac{TP}{TP+FN} \end{array}$$ (5)

From the Eqs 2 and 3, it is evident that both accuracy and F1 score can get values between [0, 1]. The better the accuracy and F1 score is the better performance.

**Fig 6:**
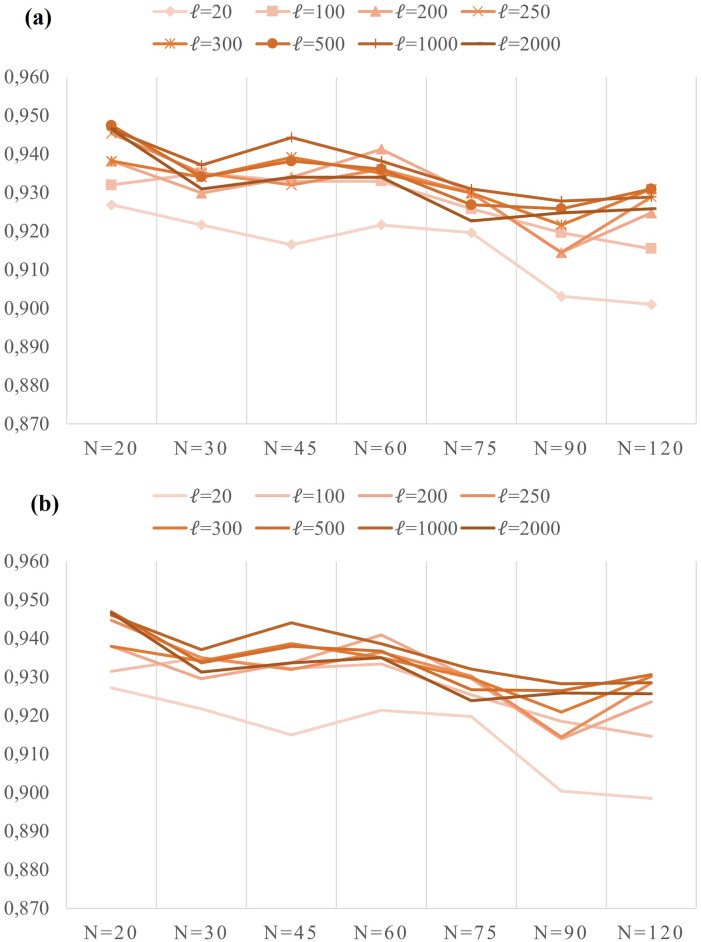
5-fold average results of test sets given in the Tables 2 and 3. (a) Accuracy (b) F1 score.

Predictions of the models are obtained by giving the videos as an input to the overall network (Fig 4). The average test accuracy and F1 score for each scenario, *s*_*i*_, are given in Tables 2 and 3. Their plots are given in Fig 6. The highest average values of test accuracy and F1 score came out as both 94.7% with 0.0132 and 0.0134 standard deviations, respectively. The (min, max) standard deviation values of Tables 2 and 3 are (0.0039, 0.0263) and (0.0032, 0.0262), respectively. The maximum observed accuracy among the 5-fold attained to 96.9%. The average values of test accuracies and F1 scores have almost been the same as validation measurements. This indicates that there was not an over or underfitting problem with the tests.

**Table 2:** 5-fold averages of the test accuracies of the proposed model for various window sizes and layer depths. The highest score is highlighted.

		Layer Depth							
		20	100	200	250	300	500	1000	2000
Window Size	20	92.7	93.2	93.8	94.5	93.8	**94.7**	94.6	94.6
	30	92.2	93.5	93	93.5	93.4	93.4	93.7	93.1
	45	91.6	93.3	93.4	93.2	93.9	93.8	94.4	93.4
	60	92.2	93.3	94.1	93.6	93.5	93.6	93.8	93.4
	75	92	92.6	93	93	93	92.7	93.1	92.3
	90	90.3	92	91.4	91.4	92.2	92.6	92.8	92.5
	120	90.1	91.5	92.5	92.9	93.1	93.1	92.9	92.6

**Table 3:** 5-fold averages of the test F1 scores of the proposed model for various window sizes and layer depths. The highest score is highlighted.

		Layer Depth							
		20	100	200	250	300	500	1000	2000
Window Size	20	92.7	93.1	93.8	94.5	93.8	**94.7**	94.6	94.6
	30	92.2	93.5	93	93.5	93.4	93.4	93.7	93.1
	45	91.5	93.2	93.4	93.2	93.9	93.8	94.4	93.4
	60	92.1	93.3	94.1	93.6	93.5	93.7	93.9	93.5
	75	92	92.5	93	93	93	92.7	93.2	92.4
	90	90.3	91.8	91.4	91.4	92.1	92.6	92.8	92.6
	120	89.9	91.5	92.4	92.8	93	93.1	92.8	92.6

The worst accuracy appears when (*N*, *ℓ*) = (120, 20). This is followed by the (90, 20) pair. (See the Table 2). The table shows an inverse correlation between the window size *N* and performance measures, accuracy, and F1 scores, for every layer depth *ℓ* with few exceptions such as (30, 1000) pair. However, the performance measurements peak when *ℓ* is around the 300-500 range. The optimal window size and layer depth parameter ranges are *N* = 20 and *ℓ* = [300–500].

We observe that shallow networks and long windows do not lead to good results from the overall results. Indeed, when the BLSTM network has 500 stacked cells fed with 20 frame-long videos, the accuracy rises to the highest. Furthermore, 250 stacked cells give as good accuracy as 500 cells. A lower window size means a lower detection time. Fortunately, the proposed method gives the highest accuracy in the smallest window size, 20. Our method reached a detection duration of 23.4 ms per frame for (*N*, *ℓ*) = (20, 250) and for the best accuracy, i.e., (*N*, *ℓ*) = (20, 500), the detection duration is 23.7 ms per frame. This shows that, contrary to [21], a window size of 20 frames, i.e., two third of a second, would only contribute a delay less than a second. This detection performance can sufficiently be considered as real-time detection.

For a typical video with 30 frames per second (fps) recording, 20 frames would take less than a second. Since the detection time is a significant concern for the first responders in the field, the proposed method significantly contributes.

In summary, a pre-trained CNN was used to extract feature maps which in turn were used to train a BLSTM network. Finally, trained CNN and BLSTM models were interconnected and given never-seen videos to show the pipeline’s real-life performance. The CNN was trained on ImageNet which does not include the fire object as a class; instead, it includes a few wild fire event-related classes which are ‘fire engine and fire truck’. We tested this pre-trained GoogLeNet on our fire images and as one expects, observed zero percent accuracy.

**Fig 7:**
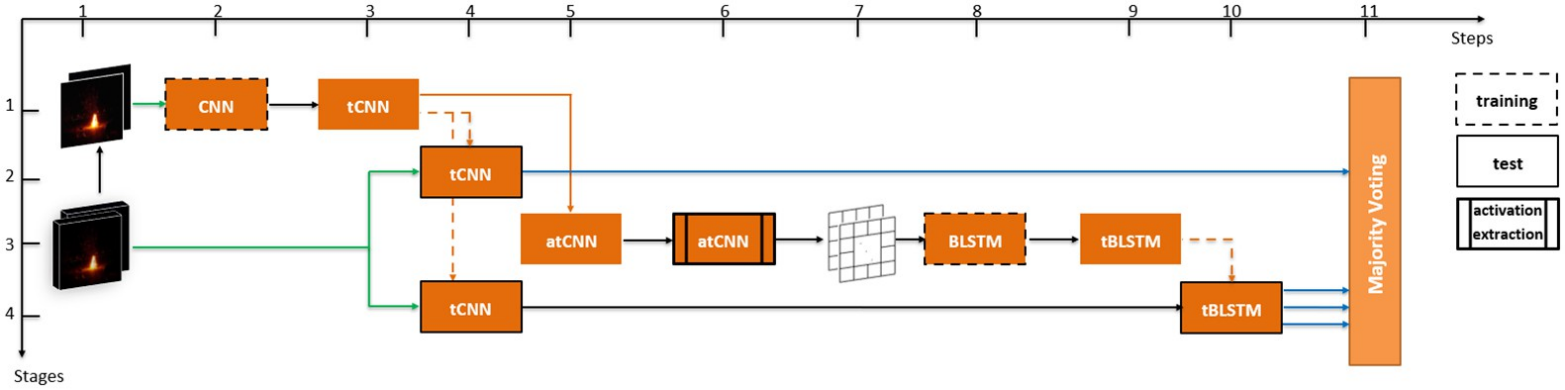
Training CNN from ground-up and employing majority voting.

Obtained results above were used to design, train, and test an improved network. The improved network used a ground-up trained CNN, tCNN, which was trained on our novel dataset rather than a pre-trained CNN (Step 2 of Stage 1 in Fig 7). Disjoint video sets for training, validation, and test were determined. In training and validation sets, one frame from each video was randomly sampled to construct an image dataset for CNN training (Step 1 of Stage 1 in Fig 7). After training the CNN, feature maps were extracted through the tCNN and they were used to re-train the BLSTM network which led to a trained BLSTM model, tBLSTM (Steps 5-9 of Stage 3). Best performing window size and layer depths from the model pipeline proposed at Section 4 were *N* = 20 and *ℓ* = [300–500], thus these parameters were chosen to be *N* = 20 and *ℓ* = {250, 500, 1000} for the improved model.

Four prediction results were acquired from the improved network. First result was obtained from tCNN at Step 4 of Stage 2 as shown in Fig 7. All frames of a test video were given to the model sequentially to see full performance of the model on the whole video. Therefore, the tCNN model gave 20 predictions per video that overall performance was computed by averaging the number of correct predictions (true positives and true negatives) over the number of all predictions. Second, third, and fourth results were obtained from tCNN+tBLSTM pipeline at Steps 4 and 10 of Stage 4 in Fig 7. Video frames were given as input to the pipeline and a single decision was obtained at the output per each layer depth tested. These four results, finally, were given as inputs to a majority voting module (Step 11) to improve the effect of spatial features on decision making. This led to the best average accuracy increase from 94.7% to 95.15%. If only the pre-trained CNN were to be used as a classifier, zero accuracy is obtained. If only the tCNN were to be used as a classifier, 94.33% accuracy is obtained. When the tCNN is coupled with the BLSTM and majority voting, the accuracy increases to 95.15%. All tests were performed in a five-fold manner as described before.

## 7 Investigating the misclassifications

To further improve the detection performance of the night-time forest fire detection algorithms, sources of misclassifications ought to be investigated. This gives an essential insight into the false classifications and might pave the way for more targeted methods.

We investigated the most frequently misclassified videos in the hope of revealing patterns that lead to wrong predictions. In that regard, the 34 most misclassified test videos, which comprise 44% of the total misclassifications, are considered sufficient for that purpose. We applied masks to these videos while preserving the original video size to see any performance improvement and possible deceptive patterns. The masks are simple black regions that cover either fire or non-fire objects depending on their shape in the scene. When an object is masked by a black region with an arbitrary shape, then that object cannot be considered in the decision process during testing a classifier model.

Adobe Premiere Pro^®^ was used to create such dynamic-shape masks and the masked videos were used for re-testing. The tests were repeated with the respective classifier that gave an error initially on the original non-masked video (As an example, see [51]).

The number of masks applied to a misclassified video can be different depending on the content of the video. Therefore, multiple test videos might be generated from a single video. In total, 76 masked videos were generated from 34 most erroneous videos. In [51], an illustration of how a mask was applied to a misclassified video is given. In the video, only two masks were used, one for fire objects and another for suspected non-fire objects as a whole.

When the masks are applied to fire videos to hide the fire portion, since no fire object is visible in it anymore, its ground truth class is converted from fire to non-fire, respectively. With the updated ground truth labels, re-tests were conducted on masked videos. This resulted in a 50.9% improvement in correctly classifying videos by using the original respective classifier models. This shows that multiple objects in a scene confuse the decision mechanism of the network. If the objects are shown to the models individually, the detection accuracy potentially changes.

[51] is an example of a missed detection. When only a fire object is left, and remaining firefighter figures are masked out, as at the 1st second in [51], then the original respective classifier model makes the new decision correctly as ‘fire.’ When only firefighters are left in the video, as at the 3rd second in [51], the original model decides ‘non-fire’ incorrectly ‘non-fire’ for the original video given in [51].

The presence of people in firefighter-like jackets (i.e., yellow jackets) also leads to missed detection as some examples are shown in [52]. In the dataset, firefighters and reporters are frequently seen in these jackets in non-fire videos and less frequently in fire videos. It indicates that the network learns ‘yellow jackets are more related to non-fire class than fire class.’ However, the network is expected to learn fire objects as fire, as well. Therefore, when ‘fire object’ and ‘yellow jacket’ are in the same scene, we observed that the fire object is in a weak appearance, not flickering, fully or partially occluded by other objects or scene borders. However, when firefighters’ jacket is not visible as yellow but mostly dark/shady as at the 2nd second or with a visible yellow jacket with a highly flickering fire object in the scene as at the 3rd second in [52], the video is successfully classified as fire.

Similarly, video in [53] was misclassified as ‘non-fire even though it is a ‘fire’ video. At the 1st second in [53], mask M1 was applied to the video, and vehicle headlights were blacked out; the ground truth label is still ‘fire.’ The same respective model could predict this video as ‘fire’ this time correctly. At the 4th second in [53], deceptive objects (headlights) were left only, and fire objects were blacked out; now, the ground truth label is ‘non-fire.’ Then the same respective model correctly classified it as ‘non-fire.’ However, this is not strong evidence that the model predicted ‘non-fire’ correctly since it was already giving the same prediction. In other words, it is a possibility that the model is replicating its incorrect decision meanwhile the ground truth label was changed. This decision pattern implies that a negative object in the scene can manipulate decision-making when a model is trained with scenes containing both negative and positive objects.

The video shown in [54] is recorded in a fire event, although there is no fire object in the scene. The trained model classified it as ‘fire’ due to vehicle headlights as numbered at the 1st second in [54]. Headlights 1, 2, and 3 are flashing while 4 is not. When flashing headlight 3 is masked out from the scene with mask M1 as shown in [54], the prediction is still incorrectly ‘fire.’ However, when all flashing lights are masked out with another mask, M2, as at the 2nd second in [54], then the model correctly predicts the scene as ‘non-fire.’ Similarly, in [55], an artificially flickering electric light was predicted as ‘fire.’ When the center and reflected halo environments were masked out separately as given at the 1st and 2nd seconds in [55], respectively, the model could predict them correctly as ‘non-fire. These two pieces of evidence strongly indicate that the flickering effect of non-fire light sources is a potential deceptive pattern for RNNs and possibly for other temporal analysis algorithms. This finding is also parallel with the reports found in the literature [17].

Flickering frequency of light at very high or very low rates has the potential to puzzle predictions. In short-range, a fire object’s flickering characteristic is apparent. If the fire object is large and strong enough in mid-range, its flickering characteristic is still evident. In long ranges, flickering characteristics become less critical in fire motion characterization. However, if the flickering rate is very high in short or mid ranges, the algorithm cannot relate its motion to average fire motion. High rate flickering occurs when a burning element contains agents with high flame propagation speed or during strong winds. Low-rate flickering occurs in matured fires that remain primarily in ember form or videos recorded/edited in slow motion [56].

Occlusion is another problem as it is for other object detection tasks, too. If other objects obstruct the fire object, this reduces the chances of correct prediction. When the indefinite form of a fire object is visible, its contour helps the algorithm in the prediction. In some cases, fire objects are obstructed in certain ways by other objects. The contour of an occluding object becomes the contour of a visible fire object. In the first two seconds of [56], it is seen that fire appears behind a window, and at the 1st and 3rd seconds in [57], fire is occluded by solid objects. Thus, the typical form of fire disappears, and it creates a fixed-contour object with fire color characteristics. It is conjectured that the algorithm incorrectly learned that fire could have a rigid shape. Therefore, this results in false positives.

When the wildfire size is immense and discharges a large amount of smoke into the environment, the light rays in the environment diffuse into smoke or fog and create a halo effect around the source [57]. Illuminated smoke leads to a diminished visible flame contour, and the nature of the fire seems smoother than it should be. In the 1st and 3rd seconds in [57], the videos were misclassified as non-fire. The fire objects in the videos have reduced visibility of flickering and smooth behavior due to dense smoke. Considering the network is trained on videos containing other light sources in dense smoke, fire objects are indistinguishable from other light sources in such foggy environments. On the other hand, a very similar video at the 2nd second in [57] includes fire objects with evident flickering. Therefore, this video was correctly classified as fire. Since fire and other light sources become less distinguishable in dense smoke environments, it prevents the network from learning fire motion. In this case, other sub-events in the scene become more important in decision making. At the 3rd second in [57], the presence of a fire-fighter in a yellow jacket has been crucial in classifying the video as non-fire.

Sometimes, objects that are not a natural light source can also resemble fire objects and mislead the network. Red fire extinguisher substance discharged from a fire-tank aircraft tricked the network 98 times in our experiments [58]. During the substances’ landing on the ground and plant area, its spread and motion resemble a fire object, and the network classifies it as fire.

In this section, we have investigated the sources of misclassifications. The tests are repeated with various modified videos to unmask the misleading parts in the contents and vulnerabilities of the networks. These additional tests showed that:

1. Multiple light sources: The decision accuracy increases if the scene has a single bright object. When both positive and negative objects appear in the scene, the model’s prediction accuracy decreases.
2. Flickering: The fire has a distinct flickering behavior. The flickering frequency diminishes as the fire gets further away. This makes it hard for the network to learn a single flickering frequency. Moreover, some other light sources, e.g., lanterns, also have a flickering nature. This is one of the unsolved challenges of these tasks.
3. Fog: A visible flame has sharp and quickly altering edges. When fog is introduced, these details, which are valuable indicators for the model, vanish.
4. Occlusion: When an object occludes the fire, a halo appears around the blocking object. This halo looks quite similar to a flame in terms of color. However, it stays still and has a somewhat different shape than fire. During the training, this might force the model to learn these features incorrectly.
5. Strobe lights: As reported, a common misclassification source is the strobe lights attached to the vehicles in the field [17]. Those lights cause problems in two respects: first, they have similar periodicity features as fires. Second, they both appear in non-fire scenes, e.g., typically in traffic and in forest fires. Those two features make the predictions less accurate if they appear in the scene.

The night-fire classification problem’s challenges can be countered by designing targeted approaches such as preprocessing filters or cascaded models. For example, in an experiment, the authors first detect and mask the possible strobe lights before the prediction [17]. These experiments intended to show and identify the challenges that exist in the night-time fire videos.

## 8 Conclusion

Night-time forest fire videos lack some important spatial information such as color, sharp edges, etc., due to the physical limitations of the camera. For some cases, distinguishing a night fire from artificial light from a single frame is highly challenging even for human-level classification. This makes the night-time fire classification harder than its daytime counterpart. To alleviate this, temporal information is incorporated in the proposed method. Thus, night fire’s natural flickering and motion behavior could be captured and involved in the analysis.

In this study, GoogLeNet + BLSTM based network architecture was used to analyze the spatio-temporal information of fire object candidates and detect fire events in night-time videos. The tests were performed for a wide range of parameter sets. The video lengths used in training and tests ranged from 20 to 120 frames. The BLSTM network depths ranged from shallow 20 layers to deep 2000 layers. It is shown in the pre-trained experiment that the shortest videos, with 20 frames and 500 BLSTM layer-deep-network gave the best parameter combinations with 94.7% accuracy and F1 score at 23.7 ms per frame. Even though the longer videos have more information and deeper networks have more adaptation capacity, they did not have the best parameters. These results were used to tune a majority voting module and the highest accuracy of 95.15% was reached.

For a typical 30 FPS video, the proposed algorithm requires less than a second to accumulate 20 frames and detect the night fire event. Since the response speed of first responders in the field is crucial, this method makes a significant contribution by reducing the response time.

The study also contains a thorough investigation and discussion of possible sources of misclassifications in night-time wildfire detection tasks. Multiple light sources, the flickering of artificial lights, strobe lights, fog, smoke, and occlusion are the primary sources of incorrect predictions.

Several problems remain unsolved. It is conjectured that by designing targeted solutions such as image preprocessing or cascaded decisions, the effects of the aforementioned false classification sources can be mitigated. Moreover, distant fires look significantly different than close ones. Instead of a unified algorithm, multiple algorithms targeting these situations separately can be designed as shown in the majority voting module.
